# Supplementary material for: The relationship between medical comorbidities and health-related quality of life among adults with type 2 diabetes: The experience of different hospitals in southern Bangladesh
Source: PLoS One. 2022 May 25;17(5):e0267713. doi: 10.1371/journal.pone.0267713 (PMC9132298; doi:10.1371/journal.pone.0267713)
Supplement: S1 Table — (DOCX) [file pone.0267713.s001.docx]

**The relationship between medical comorbidities and health-related quality of life among adults with type 2 diabetes: The experience of different hospitals in southern Bangladesh**

**Supplementary file**

**Table - Most Common multi-morbidities among diabetes patients of southern Bangladesh**

| **Multimorbidities** | **Prevalence (%)** |
| --- | --- |
| Hypertension and eye problem | 50% |
| Hypertension and heart disease | 39% |
| Hypertension and respiratory problem | 33% |
| Obesity and heart disease | 29% |
| Hypertension and obesity | 28% |
| Hypertension and kidney problem | 10% |
| Eye and neurological problem | 8% |
