# Supplementary material for: The relationship between medical comorbidities and health-related quality of life among adults with type 2 diabetes: The experience of different hospitals in southern Bangladesh
Source: PLoS One. 2022 May 25;17(5):e0267713. doi: 10.1371/journal.pone.0267713 (PMC9132298; doi:10.1371/journal.pone.0267713)
Supplement: S1 Questionnaire — (PDF) [file pone.0267713.s002.pdf]

QUESTIONNAIRE

Understating the health related quality of life and molecular basis of diabetes among the patients in the southern part of Bangladesh

Unique ID:										Date of Interview: D D M M YYYY

____

Mobile number of the subject:

Section 1: Socio-Demographic Information

1.1 Current Address: Write at least location e.g. Agrabad

1.2 Origin Address: Write at least UPAZILLA e.g. RAMU

1.3 Age (in full years): X X Years 1.4 Gender: ☐ Male ☐ Female

1.5 Religion: ☐ Islam ☐ Hindu ☐ Christian ☐ Buddhist ☐ Others

1.6 Marital status: ☐ Never married ☐ Married ☐ Widow/widower ☐ Seperated/divorced

1.7 Highest education completed: ☐Primary ☐ SSC ☐HSC ☐ Bachelor ☐ Masters ☐ Doctoral

1.8 Occupation ☐ Unemployed ☐ Service ☐ Business ☐ Retired ☐Housewife ☐ Dependent ☐ Others

1.9 Monthly family income: ☐ <20K ☐ 20K-<35K ☐ 35K-<50K ☐ 50K-<75K ☐ 75K-<1Lac ☐ >=1 Lac

Section 2: Comorbidities (Self reporting diseases)

(a) Have you ever been diagnosed with any of the following ☐Yes ☐ No (b) If yes, write age at first diagnosis (otherwise leave blank)

		YES	NO	
2.1	Please write the year of first diagnosis of diabetes			X XX X
	2.1.A Any family history of diabetes	☐	☐	☐ Mother ☐ Father ☐ grandparent ☐ Siblings
2.2	Hypertension (high blood pressure)	☐	☐	Year of first diagnosis X XX X
2.3	Heart disease (Angina, Heart Attack, Heart failure, MI, CAD)	☐	☐	Year of first diagnosis X XX X
2.4	Eye Problems (Retinopathy, visual acquity, cataract)	☐	☐	Year of first diagnosis X XX X
2.5	Kidney diseases (Kidney Failure, nephropathy)	☐	☐	Year of first diagnosis X XX X
	2.5.A Any family history of kidney disease	☐	☐	☐ Mother ☐ Father ☐ grandparent ☐ Siblings
2.6	Neurological Diseases (Stroke, TIA, paralysis, peripheral neuropathy)	☐	☐	Year of first diagnosis X XX X
2.7	Diabetic Ulcers/Diabetic foot	☐	☐	Year of first diagnosis X XX X
2.8	Cancer	☐	☐	Year of first diagnosis X XX X
	2.8.A Any family history of cancer	☐	☐	☐ Mother ☐ Father ☐ grandparent ☐ Siblings
2.9	Asthma/COPD/Bronchitis	☐	☐	Year of first diagnosis X XX X
2.10	Tuberculosis	☐	☐	Year of first diagnosis X XX X

Section 3: Medication Adherence

3.1 Do you sometimes forget to take your medicine? ☐Yes ☐ No

3.2 People sometimes miss taking their medicines for reasons other than forgetting. Thinking over the past 2 weeks, were there any days when you did not take your medicine? ☐Yes ☐ No

3.3 Have you ever cut back or stopped taking your medicine without telling your doctor because you felt worse when you took it? ☐Yes ☐ No

3.4 When you travel or leave home, do you sometimes forget to bring along your medicine? ☐Yes ☐ No

3.5 Did you take all your medicines yesterday? ☐Yes ☐ No

3.6 When you feel like your symptoms are under control, do you sometimes stop taking your medicine? ☐Yes ☐ No

3.7 Taking medicine every day is a real inconvenience for some people. Do you ever feel hassled about sticking to your treatment plan? ☐Yes ☐ No

3.8 How often do you have difficulty remembering to take all your medicine?
☐ Never/rarely ☐ Once in a while ☐ Sometimes ☐ Usually ☐ All the time

Section 4: Tobacco use

4.1 Have you ever smoked chewed or used other forms of tobacco?
☐ Never ☐ Former (stopped >6 mos.) ☐ Current (in last 6 mos.)
If currently taking tobacco,

4.2 Smoking (Cigarette/Beedi) : ☐Yes ☐ No ; if YES, write the intake frequency X X

4.3 Chewed tobacco or betel nut : ☐Yes ☐ No ; if YES, write the intake frequency X X

4.4 Used other forms of tobacco : ☐Yes ☐ No ; if YES, write the intake frequency X X

4.5 At what age you have started smoking? : X X Years of age

4.6 If current tobacco user, would you like to stop using tobacco? : ☐Yes ☐ No

Section 5: Diet and Physical activity

Now I want to ask you about fruits and vegetables that you usually eat. The card shows some local fruits and vegetables. Each picture shows the size of a serving. Think about a typical week, last week if this was typical for you.

5.1 In a typical week, on how many days do you eat fruit (show card)? X X days

5.2 How many servings of fruit do you eat on one of those days (show card)? X X servings

5.3 In a typical week, on how many days do you eat vegetables (show card)? X X days 5.4

How many servings of vegetables do you eat on those days (show card)? X X servings

If non-veg, how many servings of the following do you eat in a typical week?

5.5 Meat : X X days X X servings
5.6 Eggs : X X days X X servings
5.7 Fish : X X days X X servings
5.8 Dry fish : X X days X X servings

5.9 On average, how many meals a week do you eat that were not prepared at home? (By meal, I mean breakfast, lunch and dinner) X X meals

5.10 Do you usually add extra salt to your food before eating it? ☐Yes ☐ No

5.11 In a typical week, how many servings of processed or pre-packed foods do you eat? (e.g. biscuits, crisps) X X servings

5.12 In a typical week, how many servings of drinks (coke/fanta/sprite/7-up/energy drink) servings do you eat? X X

5.13 How much sugar (tea spoon) do you take with tea/coffee? X X tea spoon

5.14 During the past 7 days, on how many days were you physically active for a total of at least 60 minutes per day? ☐ 0 days ☐ 1 day ☐ 2 days ☐ 3 days ☐ 4 days ☐ 5 days ☐ 6 days ☐ 7 days

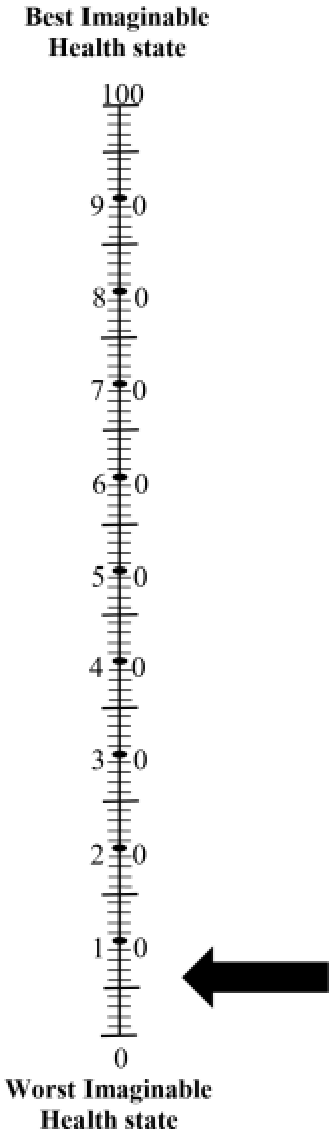

Section 6: Health Questionnaire

By placing a checkmark in one box in each group below, please indicate which statements best describe your own health state today.

6.1 **Mobility**

- ❑ I have no problems in walking about
- ❑ I have some problems in walking about
- ❑ I am confined to bed

6.2 **Self-Care**

- ❑ I have no problems with self-car
- ❑ I have some problems washing or dressing myself
- ❑ I am unable to wash or dress myself

6.3 **Usual Activities**

- ❑ I have no problems with performing my usual activities
- ❑ I have some problems with performing my usual activities
- ❑ I am unable to perform my usual activities

6.4 **Pain/Discomfort**

- ❑ I have no pain or discomfort
- ❑ I have moderate pain or discomfort
- ❑ I have extreme pain or discomfort

6.5 **Anxiety/Depression**

- ❑ I am not anxious or depressed
- ❑ I am moderately anxious or depressed
- ❑ I am extremely anxious or depressed

6.6 **Today's Health State**

To help people say how good or bad a health state is, we have drawn a scale (rather like a thermometer) on which the best state you can imagine is marked 100 and the worst state you can imagine is marked 0.

We would like you to indicate on this scale how good or bad your own health is today, in your opinion. Please do this by drawing a line from the box below to whichever point on the scale indicates how good or bad your health state is today.

Section 7: Physical Measurements

7.1 Weight: X X . X [kg] 7.2 Height: X X Feet X X Inch

7.3 Systolic BP (brachial) : X X X [mmHg] 7.4 Diastolic BP (brachial): X X X [mmHg] 7.5

Do you take hypertensive drug regularly? ☐Yes ☐ No

Section 8: Blood Tests and Physical Examination

Tests	Results	Exams	Results
8.1. FBS		8.6. Eye	
8.2. OGTT/ 2h AFB		8.7. Foot	
8.3. HbA1C		8.8. Neurological	
8.4. S. Creatinine		8.9. ECG	
8.5. SGPT		8.10. Urine Albumin	
8.11. Lipid profile	a) T. Cholesterol: b) HDL:	c)LDL:	d) TG:

8.12. CBC	a) Hb%:	b) RBC:	c)WBC:	d)
	Platelet:			

Thank you for your patience. For any query please contact with the Principal Investigator

adnan.mannan@cu.ac.bd; 01716903485

Information and consent form

Title: Understating the molecular bases of diabetes and its association with developing cancer among the diabetic patients in the Chittagong region of Bangladesh

Name of the study: Understating the molecular basis of diabetes and its association with developing cancer among the diabetic patients in the Chittagong region of Bangladesh.

Background: Diabetes is complex disorder that hampers a person/ family financially and psychologically. University of Chittagong and Chittagong Medical College are studying the polymorphism in TCF7L2 gene among the diabetic patients of Chittagong. We also want to study the correlation between socio-economic factor, lifestyle and diabetes. This study also aims to investigate the risk of cancer among diabetic patients. It will help to create awareness among the diabetes patients and to determine diabetes risk factors.

Who are the participants: Patients coming to Diabetes treatment centers of Chittagong.

Interviewer(s): Trained Research Assistant/ Associate related to this study.

Aim of the study: Investigating TCF7L2 gene polymorphism among diabetic patients of Chittagong city and their cancer risk of cancer prediction.

Method of study: Based on the consent of the subject, they will be interviewed and blood sample will be collected. It will be followed by PCR, gene sequencing and statistical analysis.

Duration: 12 months.

Benefit of the research: Creating awareness about diabetes and prediction of risk factors and cancers.

Risk: There is no extra risk due to this study.

Confidentiality: All the information will be stored in a protected server. It will only be used for research work and your name will never be disclosed.

Alternate: Participation in this study is dependent on your personal choice. Your treatment will not be affected by your decision.

I am giving consent that the information letter of this research has been provided to me and I have read it.

1. I have been informed about the method.
2. I have agreed to collect my information using audio/video (if necessary).
3. I can keep a copy of this information form.
4. I am also informed that,
    i. I might not be benefited directly from this study.
    ii. My name will not be exposed anywhere in the research.
    iii. My treatment will not be changed whether I participate in the study or not.
    iv. I can withdraw my name from the research at any point.
5. I give permission to use the result of the study with researchers.
6. I can use the result with my family and friends if I want.

Signature of the participant ………………………………….. Date ……………………. ….

Name & Signature of the interviewer…………………….………. Date ………………………..
