# Supplementary material for: The relationship between medical comorbidities and health-related quality of life among adults with type 2 diabetes: The experience of different hospitals in southern Bangladesh
Source: PLoS One. 2022 May 25;17(5):e0267713. doi: 10.1371/journal.pone.0267713 (PMC9132298; doi:10.1371/journal.pone.0267713)
Supplement: Attachment — Submitted filename: Response to reviewers.pdf [file pone.0267713.s005.pdf]

Response to reviewers

Reviewer #1:

There was an improvement in the writing and the presentation of the revised manuscript. However, some comments need to be addressed, and further edits are necessary.

For the discussion, it was stated that: "Patients with diabetes mellitus go through various degrees of HRQoL because of the chronic nature of the disease and different levels of compliance followed over time." These sentences are incomplete. Is it low or high HRQoL?

Response: Thank you for the comment. We have restated the sentences between lines 312-317.

For " HRQoL deteriorates significantly if they suffer from multiple comorbidities along with diabetes compared to the 331 patients who didn't have any comorbidity.", I think the study does not have enough evidence to lead to a conclusion on the cause and effect, i.e., chronic disease lead to a low HRQoL, and vice-versa.

Response: We have revisited the interpretation following suggestion of the learned reviewer in lines 314-316.

The bilateral correlation must be discussed in depth. For example, people with low HRQoL may neglect their health and medication, leading o further deterioration of their blood sugar levels and other chronic medical problems (hyperlipidemia, obesity, etc.).

Response: We have expanded discussion on bilateral and multilateral correlation of HRQoL and Comorbidities in Lines 317-322.

The discussion on the recommendation for intervention was also inadequate and needed elaboration. For example, how those affected individuals may improve their quality of life besides taking medication? What food is necessary to improve blood sugar and cholesterol level. How about a high fiber diet and some vegetables like ladyfinger and a low glycemic index food.

Response: We have added text highlighting the importance of healthy diet and food with low glycemic index as recommendation in lines 386-388.

Reviewer #2:

All the comments raised were satisfactorily addressed by the authors. No further query raised by this reviewer.

Response: We would like to thank the honorable reviewer for positive feedback and response. .
