# Supplementary material for: The relationship between medical comorbidities and health-related quality of life among adults with type 2 diabetes: The experience of different hospitals in southern Bangladesh
Source: PLoS One. 2022 May 25;17(5):e0267713. doi: 10.1371/journal.pone.0267713 (PMC9132298; doi:10.1371/journal.pone.0267713)
Supplement: Attachment — Submitted filename: Effect of comorbidities on health.docx [file pone.0267713.s003.docx]

**Effect of comorbidities on health-related quality of life among adults with type 2 diabetes attending different hospitals in southern Bangladesh**

Title: The title is misleading because there is an implication of the language of causality. Please rephrase the title to: “The relationship between medical comorbidities and health-related quality of life among adults with type 2 diabetes: The experience of different hospitals in southern Bangladesh”.

Overall comments:

The writing of the manuscript is good, with fair scientific merits based on the statistical methods employed. Having said that, there were some concerns of inference on the statistics of “correlation” when the author(s) mentioned that “The number of comorbidities was found to be negatively correlated with the HRQoL. “ Has any correlation study been employed in this research? The authors may change the wording of the sentences or employ the relevant statistics and discuss the findings.

Regarding the use of the statistical inference, please spell out OR as an odds ratio. The 95% CI was missing at each OR. Please insert it and standardise it through the text.

Specific comments:

Regarding abstract:

The abstract needs some revisions, as it contains the language of causality in the objective. The writing style was also required to be refined. I suggest some changes:

Objective Health-related quality of life (HRQoL) is a critical determinant to assess the severity of chronic diseases like diabetes mellitus. It has a close association with complications, comorbidities, and medical aid. This study aimed to estimate the prevalence of medical comorbidities and determine the relationship between comorbidities and HRQoL among type 2 diabetic patients of southern Bangladesh. Method: This study was a cross-sectional study conducted through face-to-face interviews using a pre-tested structured questionnaire and by reviewing patient’s health records with prior written consent. The study was conducted on 2,136 patients with type 2 diabetes attending five hospitals of Chattogram, Bangladesh, during the tenure of November 2018 to July 2019. Quality of life was measured using the widely-used index of EQ-5D that considers 243 different health states and uses a scale in which 0 indicates a health state equivalent to death and 1 indicates perfect health status. The five dimensions of the quality index included mobility, self-care, usual activities, pain or discomfort, and anxiety or depression. Results: Patients with three comorbidities and with four or more comorbidities had a higher probability of reporting “extreme problem” or “some problem” in all five dimensions of the EQ-5D index compared with those without comorbidity (OR: mobility, 3.99, 6.22; usual activity, 2.67, 5.43; self-care, 2.60, 3.95; pain or discomfort, 2.22, 3.44; anxiety or depression, 1.75, 2.45). The number of comorbidities was found to be negatively correlated with the HRQoL. Conclusion: Prevalent comorbidities were found to be the significant underlying cause of declined HRQoL. To raise diabetes awareness and for better disease management, the exposition of comorbidities in regards to HRQoL of diabetic people should be considered for type 2 diabetes management schemas.”

Regarding Introduction. The introduction is fair. Please be mindful on the punctuation in the sentences. For example, there is a comma after “To our knowledge, this…. ” I suggest the term “To the best of our knowledge,”:

“To the best of our knowledge, this study pioneers in evaluating the relationship between HRQoL and 107 comorbidities using the EQ-5D-5L instrument in the type 2 diabetes population in the southern 108 regions of Bangladesh.” It should be ‘regions’ and ‘not region’.”

It was mentioned in line 76 and 77 that, “Apart from the economical standpoints, the mere existence of diabetes also takes a psychological toll on the individual diagnosed, due to this disease’s chronic manifestations and auxiliary clinical complications.” Please change “economical” to ‘economic”. Please consider the following references for this statement, which is currently without any citations.

Mohammad Farris Iman Leong Abdullah, Hatta Sidi, Arun Ravindran, Paula Junggar Gosse, Emily Samantha Kaunismaa, Roslyn Laurie Mainland, Norlaila Mustafa, Nurul Hazwani Hatta, Puteri Arnawati, Amelia Yasmin Zulkifli, Luke Sy-Cherng Woon. How Much Do We Know About the Biopsychosocial Predictors Glycaemic Control? Age and Clinical Factors Predict Glycaemic Control, But Psychological Factors Do Not. J Diabetes Res. 2020; 2020: 2654208. Online 2020 May 1. doi: 10.1155/2020/2654208

Luke Sy-Cherng Woon, Hatta Sidi, Arun Ravindran, Paula Junggar Gosse, Roslyn Laurie Mainland, Emily Samantha Kaunismaa, Nurul Hazwani Hatta, Puteri Arnawati, Amelia Yasmin Zulkifli, Norlaila Mustafa, Mohammad Farris Iman Leong Abdullah. Depression, anxiety, and associated factors in patients with diabetes: evidence from the anxiety, depression, and personality traits in diabetes mellitus (ADAPT-DM) study. BMC Psychiatry. 2020; 20: 227. Online 2020 May 12. doi: 10.1186/s12888-020-02615-y

Luke Sy-Cherng Woon, Roslyn Laurie Mainland, Emily Samantha Kaunismaa, Paula Junggar Gosse, Arun Ravindran & Hatta Sidi. What makes poor diabetic control worse? A cross-sectional survey of biopsychosocial factors among patients with poorly controlled diabetes mellitus in Malaysia. Int J Diabetes Mellitus in Dev Countries. 2021. DOI: 10.1007/s13410-020-00918-0

Regarding methods: The methods are okay, except for the missing sampling strategy. Is there any cluster and stratified sampling performed for the two southern hospitals in Bangladesh?

Please do a correlation (Pearson/Spearman) study between all medical comorbidities and the measuring scales for quality of life.

Regarding results. The Tables are self-explanatory and relevant.

Regarding discussion. Please avoid the language of causality in your discussion as the study performed was cross-sectional.

The first paragraph stated, “Patients with diabetes mellitus go through various degrees of HRQoL because of the chronic 308 nature of the disease and different levels of compliance followed over time. HRQoL deteriorates 309 significantly if they suffer from multiple comorbidities along with diabetes compared to the 310 patients who didn’t have any comorbidity.” Based on your literature findings and clinical experience, what would the factors associated with poorer HRQoL and multiple medical comorbidities besides the confounding effect of all oxidative diseases? Could it be a personality factor? Or any comorbidities with severe mental health problems?

Luke S Woon, Hatta Sidi, Norlaila M. Factor structure of the Malay-version Generalized Anxiety Disorder-7 (GAD-7) Questionnaire among Patients with Daibetes Mellitus. Med and Health. June 2020: 15(1): 208 - 217.
